# Predictors of Conflict Among Nurses and Their Relationship with Personality Traits

**DOI:** 10.3390/nursrep15110378

**Published:** 2025-10-24

**Authors:** Ivana Jelinčić, Željka Dujmić, Ivana Barać, Nikolina Farčić, Tihomir Jovanović, Marin Mamić, Jasenka Vujanić, Marija Milić, Dunja Degmečić

**Affiliations:** 1 University Hospital Centre Osijek, J. Huttlera 4, 31 000 Osijek, Croatia; 2 Faculty of Medicine, Josip Juraj Strossmayer University of Osijek, J. Huttlera 4, 31 000 Osijek, Croatia; 3 Department of Nursing and Palliative Medicine, Faculty of Dental Medicine and Health, Josip Juraj Strossmayer University of Osijek, Crkvena 21, 31 000 Osijek, Croatia; 4 General Hospital Dr. Josip Benčević Slavonski Brod, A. Štampara 42, 35 000 Slavonski Brod, Croatia; 5 General Hospital Pakrac and Hospital of Croatian Veterans, Bolnička 74, 34 550 Pakrac, Croatia; 6 General County Hospital Požega, Osjecka 107, 34 000 Požega, Croatia; 7 Department of Psychology, Faculty of Humanities and Social Sciences, Josip Juraj Strossmayer University of Osijek, Lorenza Jagera 9, 310 00 Osijek, Croatia

**Keywords:** personality traits, nurses, conflict management, team collaboration, communication, workplace dynamics

## Abstract

**Background**: Conflicts are an inevitable part of interpersonal relationships, and personality traits influence how they are resolved. In the nursing work environment, conflicts often arise from poor communication and stress, negatively impacting nurses’ well-being and quality of care. The “Big Five” personality model highlights how traits such as extraversion, agreeableness, and emotional stability shape conflict approaches. Understanding these traits aids in developing effective conflict management strategies. This study investigates intragroup conflicts among nurses by identifying their types and examining how sociodemographic factors and personality traits predict their occurrence. The aim is to provide insights that support targeted interventions and improve team dynamics in nursing practice. **Methods**: The study was conducted as a cross-sectional analysis within the University Hospital Centre Osijek from March to August 2024, involving nurses and technicians. Data was collected using structured questionnaires with clearly defined inclusion and exclusion criteria. The questionnaire included the Process Conflict Scale, the Big Five Inventory, and a Demographic questionnaire. Appropriate statistical analyses were conducted, including descriptive statistics, normality testing with the Kolmogorov–Smirnov test, non-parametric Spearman and Point-Biserial correlations, and linear regression to examine predictors of intragroup conflicts. All assumptions for regression were met, with significance set at *p* < 0.05, and analyses were performed using JASP software version 0.17.2.1. **Results**: The research reveals significant differences among various types of team conflicts, where personality traits such as neuroticism increase, while conscientiousness decreases conflicts. The professional competence of respondents also positively correlates with logistical conflicts, and personality explains the variance in conflicts among nurses. **Conclusions**: Intragroup conflicts among nurses, particularly task-related, stem from communication issues and high care standards. Neuroticism negatively affects team dynamics, while conscientiousness can reduce conflicts but may also lead to disagreements if expectations are unmet. Education on conflict management and clearly defined roles can improve teamwork and quality of care.

## 1. Introduction

### 1.1. Background

Conflicts are an inherent part of interpersonal relationships, influenced by individual personality traits. Nurses’ working conditions, including their environment, tasks, and psychological states, often expose them to workplace conflicts [1]. Studies show that conflicts among nurses are frequent, especially in hospital settings [2,3,4,5,6]. These conflicts negatively impact nurses’ health and well-being, disrupt organizational functioning, and compromise patient care quality [3,7]. Common causes include poor communication, work-related stress, and disagreements over priorities or task allocation [8,9]. Such conflicts diminish motivation, job satisfaction, and team effectiveness while increasing stress, which can lead to higher turnover and reduced engagement in teamwork. Consequently, this environment may result in more medical errors, lower treatment quality, and decreased patient trust in healthcare services [10,11]. For these reasons, effective management of interpersonal relationships and conflict prevention becomes crucial, not only for the health and well-being of employees but also for preserving the quality of healthcare.

### 1.2. Literature Review

Within nursing teams, effective communication is essential for providing quality care. However, it is often hindered by work overload and the lack of regular meetings, which can lead to mistakes. To improve organizational safety and enhance collaboration and engagement among healthcare professionals, it is important to advance communication processes and optimize human resource management [12,13]. These interpersonal communication skills in nursing may be influenced by individual personality traits, and in recent years, the Big Five personality model has gained attention as a useful framework for understanding such characteristics. The “Big Five” personality model consists of five key dimensions: extraversion, agreeableness, conscientiousness, emotional stability, and openness to experience [14]. Individuals with high extraversion are typically sociable and communicative, willing to confront conflicts openly. Their energy and confidence can be beneficial in negotiations, but their direct approach may sometimes appear aggressive, complicating constructive dialogue. Agreeableness is manifested in the effort to resolve conflicts peacefully. People with high levels of agreeableness often prefer compromise and empathy, avoiding conflicts to maintain harmonious relationships. However, this can sometimes mean neglecting their own needs to preserve peace. Conscientious individuals approach conflicts with an organized plan. Their tendency for detailed thinking and analysis can contribute to the development of long-term and thoughtful solutions. However, their need for structure can hinder their adaptability to fast and unpredictable situations. Emotionally stable individuals remain calm under pressure, managing conflicts rationally. In contrast, those who are emotionally unstable may react with greater intensity, making it difficult for them to face challenges. Individuals open to experience bring creativity and flexibility to conflict resolution. They are often willing to explore new ideas and consider diverse perspectives, which can lead to innovative solutions and adaptability to change. Understanding how personality traits influence behavior in conflicts can help us develop more effective strategies for resolving these situations.

### 1.3. Study Aims and Significance

This study aims to comprehensively examine intragroup conflicts among nurses by identifying the types of conflicts that commonly occur within nursing teams—such as relationship, task, logistical, and contribution conflicts—and by exploring the predictors of these conflicts through an analysis of sociodemographic variables (e.g., age, gender, education, work experience) and personality traits based on the Big Five model. Furthermore, the research investigates the relationships between these conflicts and individual characteristics, with the goal of providing insights that can inform targeted interventions, enhance team dynamics, and ultimately improve the quality and effectiveness of nursing practice. It examines the types of conflicts that arise within nursing teams, including relationship, task, logistical, and contribution conflicts. In the context of nursing teams, conflict can take various forms, each with distinct causes and impacts on team dynamics and patient care. Task conflict refers to disagreements among team members about clinical decisions, patient care plans, or approaches to treatment. These conflicts involve differences in opinions or ideas related to the work itself and, when managed constructively, can lead to improved decision-making and outcomes. Importantly, task conflict does not typically involve personal animosity. In contrast, relational conflict arises from interpersonal tensions or incompatibilities among team members. It may involve feelings of frustration, irritation, or hostility, and often stems from personal issues rather than work-related disagreements. This type of conflict can significantly disrupt teamwork, lower job satisfaction, and negatively affect the work environment and quality of care. Logistical conflict involves challenges in coordinating tasks and managing shared resources. Common sources include disputes over shift scheduling, workload distribution, and delegation of responsibilities. If unresolved, logistical conflicts can reduce team efficiency and morale. Finally, contributional conflict occurs when team members perceive an imbalance in individual contributions. This may involve concerns that some colleagues are not fulfilling their responsibilities, are less reliable, or that others’ efforts are not being appropriately recognized. For example, if a nurse consistently arrives late or avoids shared duties, it can lead to frustration and feelings of inequity among the team. Such conflicts can harm collaboration, diminish trust, and ultimately compromise patient care.

This study aims to fill that theoretical and empirical gap by adopting a multidimensional approach to understanding conflict—not only by examining its manifestations and management strategies but also by exploring the influence of individual characteristics (e.g., personality traits based on the Big Five model) and sociodemographic variables such as age, gender, education level, and work experience. This approach offers a more nuanced understanding of how personal and contextual differences shape perceptions of conflict and the strategies employed to manage it, thereby representing the unique contribution of this study to the existing body of literature.

### 1.4. Practical Contribution of the Study

The practical contribution of this study lies in the potential implementation of targeted interventions aimed at improving the work climate and reducing the frequency of conflicts within healthcare teams. Training in emotional intelligence serves as a critical tool for enhancing self-awareness, emotional regulation, and effective interpersonal management, which is particularly important in the high-stress and demanding environment of healthcare institutions. Such training equips employees with the skills necessary for constructive conflict resolution, tension reduction, and strengthened collaboration.

Moreover, the establishment of clear institutional communication protocols contributes to the standardization and transparency of communication within the organization. This reduces misunderstandings and conflicts arising from unclear or inadequate information exchange. Effective communication protocols ensure timely and accurate information flow, which is essential for coordinating teamwork and maintaining a professional work environment.

Additionally, the introduction of support systems and supervision provides ongoing assistance to staff in coping with professional challenges and emotional burdens. Regular supervisory sessions facilitate reflection on work experiences, support the development of professional competencies, and reduce the risk of burnout, all of which contribute to a healthier work atmosphere and improved quality of healthcare delivery.

The integration of these approaches into routine healthcare practice can significantly enhance the work environment, increase employee satisfaction, and consequently improve the quality of patient care.

## 2. Materials and Methods

Cross-sectional research was conducted. The research was planned to last five months, from March to August 2024. Data were collected through self-administered questionnaires, with completion times ranging between 15 and 20 min, thereby ensuring efficient data collection with minimal participant burden. The data collection process was conducted by the principal investigator, Ivana Jelinčić. Data collection took place within the hospital setting, specifically in separate rooms designated within each organizational unit of the University Hospital Centre Osijek, to ensure participant privacy and concentration during questionnaire completion. Participants were informed about the study through a formal meeting organized specifically for this purpose. The response rate was consistent with the predefined inclusion and exclusion criteria. Only those individuals who wished to participate but did not meet the specified criteria were excluded from the study. Additionally, 47 participants were excluded due to incomplete completion of the questionnaire. The remaining 394 participants, who met the inclusion criteria and fully completed the questionnaire, were included in the study described.

Inclusion criteria stipulated that participants must have a minimum of six months of continuous employment within the same organizational unit. Exclusion criteria included nurses and technicians who had been on sick leave or had changed their job position within the University Hospital Centre Osijek in the six months preceding the study. These criteria were established to ensure a homogeneous sample and to enhance the relevance and validity of the collected data in the context of this research. The study received ethical approval from the Committee for Ethical and Professional Issues of Nurses and Technicians at the University Hospital Centre Osijek (Approval Number: R1-4386-3/2023), ensuring adherence to ethical standards and participant rights throughout the research process. Appropriate statistical methods were employed for data analysis. Descriptive statistics were used to summarize the distribution of variables. The Kolmogorov–Smirnov test was applied to assess the normality of numerical variables, which revealed significant deviations for all numerical variables (age, length of service, personality traits, and intragroup conflicts). Consequently, Spearman’s non-parametric correlations were used to examine relationships between variables. The association between gender and intragroup conflicts was assessed using Point-Biserial correlations. Median and interquartile ranges were reported as measures of central tendency. To investigate predictors of intragroup conflicts, linear regression analysis was conducted, including variables previously identified as significantly correlated. Assumptions for linear regression were tested and met: independence of residuals was confirmed by Durbin–Watson statistics (ranging from 1.687 to 2.010), multicollinearity was assessed with variance inflation factor (VIF) values between 1.006 and 1.926 indicating low to moderate multicollinearity, and residuals were normally distributed and homoscedastic. Statistical significance was set at *p* < 0.05. Data processing was performed using JASP statistical software, version 0.17.2.1 (Department of Psychological Methods, University of Amsterdam, Amsterdam, The Netherlands). This comprehensive methodological description aims to ensure transparency and rigor in the research process, addressing concerns related to instrument usage, data collection procedures, data analysis, and ethical considerations.

### 2.1. Participants

This research included nurses and technicians of all education levels, encompassing those employed as general nursing staff, bachelor’s degree holders in nursing, as well as master’s level nurses. This diversity in educational profiles allowed for a comprehensive insight into the practices and experiences of nurses and technicians, ensuring that the research results were representative and relevant to various aspects of healthcare within the institution. The research was conducted within the University Hospital Centre Osijek which consists of 21 organizational units (departments and clinics). The total number of participants in the study was 394.

### 2.2. Instruments

For the purposes of this study, two instruments were used: the adapted version of the Intragroup Conflict Scale (originally the Process Conflict Scale) and the Big Five Inventory (BFI 44). Written permission to use the Intragroup Conflict Scale was obtained from the original author, ensuring the legality and appropriateness of its application in our research. The BFI 44 is publicly available and free to use; thus no additional copyright permission was required.

Intragroup Conflict Scale (adapted version of the “Process Conflict Scale”) [15]—This scale, containing 13 items describing different forms and sources of conflict, was employed in the research. Participants rated how often the specified events or conflicts occurred in their group on a five-point scale. Lower scores on the scale indicated less frequency (1—never; 2—rarely; 3—sometimes; 4—often; 5—always). Data collected from the Croatian sample confirmed the factorial structure of the original questionnaire, which includes four factors: relationship conflict, task conflict, logistical conflict, and contribution conflict. These factors are further divided into items: the first four items relate to relationship conflict; the fourth, fifth, and sixth items represent task conflict; the seventh, eighth, and ninth items pertain to logistical conflict; and the eleventh, twelfth, and thirteenth items relate to contribution conflict. The Intragroup Conflict Scale used in this study was developed based on a multidimensional approach to understanding conflict in team settings. Its construction builds upon a well-established model that differentiates between task conflict, relationship conflict, and process conflict. Given the conceptual and empirical ambiguities surrounding process conflict, the authors further operationalized this dimension by dividing it into two distinct subtypes: logistical conflict and contribution conflict. This refinement enables a more precise understanding of various sources of team conflict, particularly those related to task coordination, the distribution of responsibilities, and individual contributions to collective work.

The instrument is grounded in solid theoretical foundations, capturing affective, cognitive, and procedural aspects of conflict. Developed and validated through a combination of qualitative and quantitative studies on a large sample, the scale demonstrates a robust four-factor structure. All subscales exhibit high internal consistency, with Cronbach’s alpha coefficients of 0.91 for relationship conflict, 0.83 for task conflict, 0.84 for logistical conflict, and 0.92 for contribution conflict. Confirmatory factor analysis further supports the model’s excellent fit to empirical data. Additionally, construct validity was assessed via correlations with variables such as group atmosphere, with results showing expected negative associations between perceived conflict and positive aspects of team functioning, including trust, cohesion, and mutual respect.

The Intragroup Conflict Scale demonstrates strong psychometric properties. Given the high prevalence of group and team work—and consequently the inevitable conflicts arising within such settings—this scale has been widely applied. The scale was translated into Croatian using the back-translation method, with any discrepancies resolved through consultation with an English language expert. The Croatian validation study was conducted on a sample of 835 educational professionals in the Republic of Croatia.

To verify the expected four-factor structure, a confirmatory factor analysis (CFA) was performed using AMOS 26, employing the Maximum Likelihood (ML) estimation method. Model fit was assessed using the following indices: Tucker–Lewis Index (TLI), Comparative Fit Index (CFI), Root-Mean-Square Error of Approximation (RMSEA), and Standardized Root-Mean-Square Residual (SRMR). The resulting values were: TLI = 0.96, CFI = 0.97, RMSEA = 0.06, and SRMR = 0.03.

Therefore, the data collected from the Croatian sample confirm the factorial structure of the original questionnaire, encompassing four factors: relationship conflict, task conflict, logistical conflict, and contribution conflict. The scale is broadly applicable across various professional fields and organizational settings characterized by the presence of larger groups or teams. In such environments, interpersonal and task-related conflicts are common and often arise naturally as a result of group dynamics. Therefore, this instrument serves as a valuable tool for assessing and understanding conflict dimensions where group interactions and collaboration play a significant role, facilitating targeted conflict management and intervention strategies.

In terms of applicability to nursing professionals, it can be argued that all four conflict dimensions are highly relevant to the healthcare context. Nursing work is inherently collaborative and requires a high level of coordination, communication, and role clarity, all of which can give rise to conflict—whether related to task organization or interpersonal dynamics. Conflicts stemming from unequal contribution, ambiguous role expectations, and differing approaches to task execution are frequent in nurses’ daily practice. Therefore, this scale appears to be highly applicable in nursing settings, requiring only minor linguistic or contextual adjustments. The clear differentiation among conflict types allows for a more accurate diagnosis of team dynamics and offers a valuable diagnostic and research tool for healthcare organizations aiming to improve team effectiveness and workplace climate.

Contributory conflict refers to disagreements concerning the coordination of individuals within group work, including the contributions (or lack thereof) of team members and disruptive behaviors (e.g., lack of preparation), which negatively impact team effectiveness.

Big Five Inventory (BFI 44)—For personality trait analysis, the Big Five personality model was used, consisting of 44 items divided into five dimensions: extraversion (8 items), conscientiousness (9 items), agreeableness (9 items), neuroticism (8 items), and openness (10 items). This self-assessment questionnaire includes short statements such as “I am a person who worries a lot.” Responses were rated on a Likert scale from 1 to 5, where 1 represents strong disagreement and 5 strong agreement. Results are presented as the arithmetic mean of responses for each of the five factors. The BFI is considered psychometrically valid as it comprehensively covers all five personality dimensions. Internal consistency, measured by Cronbach’s alpha coefficient, typically ranges from 0.75 to 0.90, with an average above 0.80. Test–Retest reliability after three months ranges from 0.80 to 0.90, with an average of 0.85, and it shows high convergent validity. The scale is free and available for public use, so no copyright permission is needed for its use.

Structured Demographic Questionnaire—This questionnaire included questions regarding age, gender, length of employment, place of residence, and educational qualifications.

## 3. Results

Of the total number of nurses who participated in the study, the majority were female, totaling 330 (83.8%), with completed high school education, totaling 236 (59.9%). Most of them lived in urban areas, in total 230 (58.4%) (Table 1).

To determine the differences in intragroup conflicts, Friedman’s test for dependent samples was conducted. The results showed a significant difference in the values of intragroup conflicts (χ^2^ = 69.736; df = 3; *p* < 0.001). The task conflict has a significantly higher value compared to relationship conflict (Conover Bonf; *p* < 0.001), contribution conflict (Conover Bonf; *p* < 0.001), and logistical conflict (Conover Bonf; *p* < 0.001) (Table 2).

Table 3 displays the descriptive statistics of the personality traits of nurses (Table 3).

The results indicated a significant low positive correlation between relationship conflict and the personality trait of neuroticism (ρ = 0.159; *p* = 0.001), and a negative correlation with the personality traits of agreeableness (ρ = −0.212; *p* = 0.001), extraversion (ρ = −0.185; *p* < 0.001), and conscientiousness (ρ = −0.165; *p* = 0.001). This means that as the personality trait of neuroticism increases, relationship conflicts decrease, and as the personality traits of extraversion, agreeableness, and conscientiousness decrease, relationship conflict increases, and vice versa. Task conflict was found to have a low negative correlation with the personality trait of conscientiousness (ρ = −0.100; *p* = 0.048) and a low positive correlation with neuroticism (ρ = 0.147; *p* = 0.003). This means that as the personality trait of conscientiousness decreases and neuroticism increases, task conflict increases, and vice versa. Logistical conflict showed a low negative correlation with the personality trait of conscientiousness (ρ = −0.103; *p* = 0.040), meaning that as the personality trait of conscientiousness increases, logistical conflict decreases, and vice versa. Contribution conflict had a low negative correlation with the personality trait of agreeableness and a low positive correlation with neuroticism, indicating that as the personality trait of agreeableness decreases and neuroticism increases, contribution conflict increases, and vice versa (Table 4).

The results indicated that logistical conflict is positively correlated with the educational qualification of the respondents (ρ = 0.128; *p* = 0.011), meaning that as the educational qualification increases, logistical conflict also increases, and vice versa (Table 5).

To determine the predictors of relationship conflict, a linear regression analysis was conducted. The model included variables that were significantly associated in the previous analysis: the personality traits of extraversion, agreeableness, conscientiousness, and neuroticism. The results showed that these variables significantly explain 6.6% of the variance in relationship conflict (AR^2^ = 0.066; *p* < 0.001). The personality trait of extraversion was found to be significant (*p* = 0.036). The β coefficient indicates that the personality trait of extraversion negatively contributes to relationship conflict among nurses (Table 6).

To determine the predictors of task conflict, a linear regression analysis was conducted. The model included variables that were significantly associated in the previous analysis: the personality traits of conscientiousness and neuroticism. The results showed that these variables significantly explain 2.6% of the variance in task conflict (AR^2^ = 0.026; *p* = 0.002). The personality trait of neuroticism was found to be significant (*p* = 0.029). The β coefficient indicates that the personality trait of neuroticism positively contributes to task conflict among nurses (Table 7).

To determine the predictors of logistical conflict, a linear regression analysis was conducted. The model included variables that were significantly associated in the previous analysis: the personality trait of conscientiousness and educational qualification. The results showed that these variables significantly explain 2.8% of the variance in logistical conflict (AR^2^ = 0.028; *p* = 0.001). The personality trait of conscientiousness (*p* = 0.006) and educational qualification (*p* = 0.009) were found to be significant. The β coefficient indicates that the personality trait of conscientiousness negatively contributes to logistical conflict, while educational qualification positively contributes to logistical conflict among nurses (Table 8).

To determine the predictors of contribution conflict, a linear regression analysis was conducted. The model included variables that were significantly associated in the previous analysis: the personality traits of agreeableness and neuroticism. The results showed that these variables significantly explain 2.4% of the variance in contribution conflict (AR^2^ = 0.024; *p* < 0.003). The personality trait of neuroticism was found to be significant (*p* = 0.006). The β coefficient indicates that the personality trait of neuroticism positively contributes to contribution conflict among nurses (Table 9).

## 4. Discussion

### 4.1. Summary of Findings

This research investigates intragroup conflicts among nurses, focusing on the predictors of different types of conflicts and their relationship with sociodemographic variables and personality traits. The results indicate significant variations in the intensity of intragroup conflicts, with task conflict identified as the most pronounced compared to relationship conflict, contribution conflict, and logistical conflict. These differences imply that nurses face greater challenges in executing work tasks, which can negatively affect team cooperation and the overall quality of healthcare [13]. A low positive correlation was found between neuroticism and relationship conflicts, suggesting individuals with higher neuroticism may develop emotional management skills to reduce interpersonal tensions.

Although the majority of the literature suggests that neuroticism is typically associated with increased conflict due to a predisposition toward negative emotions and emotional reactivity, several factors may explain the observed positive correlation between neuroticism and conflict reduction in our study.

Individuals with higher levels of neuroticism may be more aware of their emotional responses and potential conflicts, which could lead them to develop self-regulation or conflict avoidance strategies to mitigate negative outcomes. In other words, heightened sensitivity to stress might motivate these individuals to actively seek ways to reduce conflicts, such as compromise, withdrawal, or seeking support, thereby decreasing the frequency or intensity of conflicts. Second, the specific context or cultural setting in which the study was conducted may significantly influence these relationships. For instance, in collectivist or highly structured work environments, neurotic individuals may be motivated to maintain team harmony due to fear of conflict consequences, resulting in reduced overt conflict expression. Third, it is possible that some measures or interpretations of the conflict scale in this study capture conflicts in terms of their visibility or escalation, while neurotic individuals, despite experiencing internal tension, may actively work to suppress or minimize conflicts. These findings highlight the need for further research to explore the mechanisms through which neuroticism influences conflict, including potential mediators such as coping strategies, workplace stress, and organizational climate. Although neuroticism is usually associated with increased conflict, our study found it linked to conflict reduction. This may be due to greater emotional self-awareness, conflict management strategies like avoidance or compromise, cultural or organizational factors promoting harmony, or the difference between internal tension and outward conflict behaviors.

Furthermore, lower levels of agreeableness, extraversion, and conscientiousness were associated with greater conflicts in team environments [16,17,18,19,20]. Personality traits such as extraversion, agreeableness, conscientiousness, and neuroticism together explained 6.6% of the variance in conflict, with conscientiousness and neuroticism explaining smaller but important proportions of task and relationship conflicts [21,22,23].

### 4.2. Interpretation

The prominence of task conflict likely reflects the dynamic and stressful nursing work environment, where different approaches to task execution led to disagreements and tension. Communication problems and high demands for quality care increase pressures, intensifying task-related conflicts [13]. Nurses with higher neuroticism might cope with stress by managing emotions, but those with high neuroticism and low extraversion may struggle more in critical situations [16]. Low agreeableness and extraversion can reduce empathy and communication quality, leading to increased risk of errors [17]. Conscientious nurses’ organized and proactive approach helps reduce task and logistical conflicts, contributing to better team efficiency [18]. Contribution conflicts arise more among those with low agreeableness and high neuroticism, as these traits hinder cooperation and increase stress [19,20]. The positive correlation between professional education and logistical conflict may reflect increased responsibilities and expectations that create disagreements [13].

### 4.3. Comparison with Previous Studies

Our findings align with prior research demonstrating that task conflict is the most common intragroup conflict type among nurses [12]. The observed correlations between personality traits and conflict types are consistent with earlier studies highlighting the protective role of agreeableness and extraversion in conflict resolution, and the detrimental effect of neuroticism [16,17,21]. The finding that conscientiousness relates to fewer task and logistical conflicts also supports previous research emphasizing its role in effective task management [18]. The complex relationship between neuroticism, extraversion, and conflict management echoes results from other studies emphasizing the need to understand personality interactions in workplace dynamics [22,23].

### 4.4. Practical Implications

Given that personality traits explain a measurable portion of conflict variance, healthcare organizations should consider personality assessments when developing team building and conflict management programs. Training aimed at enhancing emotional intelligence, communication skills, and constructive use of extraversion could reduce interpersonal tensions and improve collaboration [21,22]. Providing targeted conflict management education is particularly important for nurses with higher neuroticism and lower agreeableness or extraversion to prevent negative impacts on teamwork and patient care. Furthermore, recognizing the impact of professional education on logistical conflicts suggests the need for clear role definitions and conflict resolution training tailored to experience levels [13]. Overall, optimizing human resource management and communication processes can foster a healthier work environment and improve healthcare quality.

Numerous scholars highlight that successful conflict management relies on the use of well-rounded strategies aimed at both preventing and addressing interpersonal tensions within healthcare teams. Essential elements of these approaches include maintaining clear and consistent communication, offering ongoing training in conflict resolution, fostering a collaborative and inclusive team environment, and implementing targeted interventions to support constructive handling of conflicts. These actions are considered vital for reducing conflict and promoting a psychologically safe, cohesive, and effective team dynamic [24,25,26,27,28]. The multifaceted nature of conflict structures renders the processes of identifying, analyzing, and effectively managing conflicts inherently complex. Addressing such challenges within professional environments—particularly in the healthcare sector—necessitates a high level of expertise and the development of specific competencies. A comprehensive understanding of the underlying causes and dynamics of conflict, the ability to distinguish between its various components (such as interpersonal, organizational, or systemic factors), and the capacity to engage in structured and strategic resolution efforts are all essential elements of effective conflict management. These capabilities are considered integral to the professional competency profile of healthcare organization managers and public health administrators, as they play a pivotal role in maintaining organizational stability, enhancing team performance, and ensuring the delivery of high-quality healthcare services [29,30].

### 4.5. Study Limitations

This study explains a modest proportion of the variance in intragroup conflicts (e.g., 6.6% for overall conflict), indicating that other unmeasured factors likely play significant roles. The cross-sectional design limits causal inferences between personality traits and conflict types. Additionally, self-reported data may introduce bias due to subjective perceptions. The study sample, although large, is specific to certain healthcare settings, which may limit generalizability. Future research should incorporate longitudinal designs and diverse populations to validate and extend these findings.

### 4.6. Future Directions

Improving team dynamics is essential for enhancing efficiency and quality in healthcare settings. Providing education on conflict management equips staff with the skills to recognize and resolve disagreements constructively. Developing emotional intelligence further supports individuals in understanding and regulating their own and others’ emotions, thereby facilitating healthier interpersonal interactions. Additionally, clearly defining roles and responsibilities helps minimize misunderstandings and fosters a more harmonious and productive work environment. Emphasizing communication skills is also critical, as it directly impacts the quality of patient care.

Understanding conflict predictors, including sociodemographic factors and personality traits, is crucial for identifying sources of tension among nursing staff. This knowledge enables targeted interventions to mitigate the adverse effects of conflict and improve workplace atmosphere.

Future studies could explore additional psychological and organizational factors influencing conflict, such as emotional intelligence, leadership styles, and organizational culture. Longitudinal research would help clarify causal relationships and changes in conflict dynamics over time. Investigating intervention effectiveness targeting personality-informed conflict management in nursing teams may offer practical benefits. Moreover, integrating qualitative approaches could provide deeper insight into the lived experiences of nurses facing intragroup conflicts, enriching understanding and informing tailored strategies.

In addressing conflicts among nurses, formative strategies such as simulation-based training have proven to be effective approaches for developing awareness, adaptability, and related competencies. These training programs foster the development of key personality traits and communication skills essential for effective teamwork in healthcare settings. By engaging nurses in realistic scenarios, simulation training facilitates reflective learning and practical problem-solving, which contribute to improved conflict management and collaboration within teams. The implementation of such formative approaches can significantly reduce interpersonal tensions and enhance overall team performance in the demanding healthcare environment.

## 5. Conclusions

Intragroup conflicts among nurses are common, with task-related conflicts being the most frequent. These arise mainly from communication issues and high care demands, affecting teamwork and patient outcomes.

Personality traits affect conflict dynamics. High neuroticism is linked to emotional instability and poor decision-making, increasing conflicts. Conscientiousness reduces task conflicts but may cause disagreements when expectations differ.

Conflict management training and emotional intelligence development are crucial. Clear roles, better scheduling, and team activities reduce stress and misunderstandings. Self-awareness helps nurses recognize their role in conflicts. Ongoing training improves coping skills, care quality, and job satisfaction.

Limitations include sample size, response bias, and specific healthcare settings, limiting generalizability. Cultural and socioeconomic factors may also affect results. Despite this, conflict management is vital for improving nursing environments.

## Figures and Tables

**Table 1:** Sociodemographic characteristics of participants.

		n (%)
**Gender**	female	330 (83.8)
	male	64 (16.2)
**Educational** **Qualification**	high school	236 (59.9)
	The undergraduate university study programme of Nursing	90 (22.8)
	the graduate university study programme of Nursing	67 (17)
	Postgraduate programme	1 (0.3)
**Place of Residence**	city	230 (58.4)
	suburban area	44 (11.2)
	rural settlement	120 (30.5)
	Me (IQR)	
**Age**	41 (29–50)	
**Length of** **Employment**	20 (8–30)	

Note: n—number of participants; %—percentage.

**Table 2:** Descriptive statistics of intragroup conflicts.

	Me (IQR)	χ^2^	*p* *
relationship conflict	2.5 (2–3)	69,736	**<0.001**
task conflict	2.66 (2–3)		
logistical conflict	2.33 (2–3)		
contribution conflict	2.33 (2–3)		

Note: Me—Median; IQR—Interquartile range; χ^2^—Value of Friedman’s Test; * *p*—Statistical significance.

**Table 3:** Descriptive statistics of personality traits.

	Me (IQR)
Extraversion	27 (25–30)
Agreeableness	35 (32–37)
Conscientiousness	34 (32–37)
Neuroticism	21 (18–23)
Openness to experience	33 (30–35)

Note: Me—Median; IQR—Interquartile range.

**Table 4:** Association of intragroup conflicts with personality traits.

		Extraversion	Agreeableness	Conscientiousness	Neuroticism	Openness
relationship conflict	ρ	−0.185	−0.212	−0.165	0.159	−0.026
	*p* *	**<0.001**	**<0.001**	**0.001**	**0.001**	0.612
task conflict	ρ	−0.049	−0.092	−0.100	0.147	0.054
	*p* *	0.331	0.068	**0.048**	**0.003**	0.281
logistical conflict	ρ	−0.052	−0.079	−0.103	0.096	0.047
	*p* *	0.307	0.119	**0.040**	0.057	0.353
contribution conflict	ρ	−0.076	−0.110	−0.093	0.154	0.036
	*p* *	0.134	**0.028**	0.064	**0.002**	0.481

Note: *p*—Statistical significance; ρ—Spearman’s correlation coefficient; *—Spearman correlations.

**Table 5:** Association of intragroup conflicts with sociodemographic variables.

		Gender ^†^	Age	Educational Qualification	Length of Service	Place of Residence
relationship conflict	ρ	0.016	0.067	0.056	0.078	−0.008
	*p* *	0.757	0.182	0.266	0.120	0.873
task conflict	ρ	0.060	−0.043	0.082	−0.021	−0.070
	*p* *	0.234	0.393	0.102	0.675	0.167
logistical conflict	ρ	0.058	0.061	0.128	0.086	−0.047
	*p* *	0.254	0.225	**0.011**	0.088	0.347
contribution conflict	ρ	−0.013	0.018	0.053	0.049	−0.007
	*p* *	0.797	0.721	0.291	0.335	0.888

Note: *p*—Statistical significance; ρ—Spearman’s correlation coefficient; *—Spearman correlations; ^†^—Point-Biserial correlations.

**Table 6:** Predictors of relationship conflict.

Model	Standardized Coefficients		t	*p*	Adjusted R^2^
	β				
1	(Constant)		6.652	<0.001	0.066
	extraversion	−0.122	−2.108	**0.036**	
	agreeableness	−0.095	−1.458	0.146	
	conscientiousness	−0.065	−0.954	0.341	
	neuroticism	0.051	0.885	0.377	

Note: β—regression coefficient; t—the size of the difference relative to the variation in the sample data; *p*—statistical significance; Adjusted R^2^—coefficient of determination.

**Table 7:** Predictors of task conflict.

Model		Standardized Coefficients	t	*p*	
		β			Adjusted R^2^
1	(Constant)		6.365	<0.001	0.026
	conscientiousness	−0.085	−1.552	0.121	
	neuroticism	0.120	2.185	**0.029**	

Note: β—regression coefficient; t—the size of the difference relative to the variation in the sample data; *p*—statistical significance; Adjusted R^2^—coefficient of determination.

**Table 8:** Predictors of logistical conflict.

Model	Standardized Coefficients		t	*p*	
	β				Adjusted R^2^
1	1 (Constant)		10.651	<0.001	0.028
	educational qualification	0.131	2.635	**0.009**	
	conscientiousness	−0.138	−2.765	**0.006**	

Note: β—regression coefficient; t—the size of the difference relative to the variation in the sample data; *p*—statistical significance; Adjusted R^2^—coefficient of determination.

**Table 9:** Predictors of contribution conflict.

Model		Standardized Coefficients	t	*p*	
		β			Adjusted R^2^
1	(Constant)		3.661	<0.001	0.024
	agreeableness	−0.032	−0.571	0.568	
	neuroticism	0.154	2.781	**0.006**	

Note: β—regression coefficient; t—the size of the difference relative to the variation in the sample data; *p*—statistical significance; Adjusted R^2^—coefficient of determination.

## Data Availability

The raw data supporting the conclusions of this article will be made available by the authors on request.

## Public Involvement Statement

No public involvement in any aspect of this research.

## Guidelines and Standards Statement

This manuscript was drafted against the Strengthening the Reporting of Observational Studies in Epidemiology (STROBE) guidelines for observational research.

## Abbreviation

The following abbreviation is used in this manuscript:

BFI	Big Five Inventory
